# Perceived organizational support and self-efficacy as mediating factors between job burnout and emergency response competence among new nurses: a study based on emergency departments and intensive care units

**DOI:** 10.3389/fpubh.2026.1775808

**Published:** 2026-04-08

**Authors:** Xiao Jie Bian, Su Yu Gao, Shi Qing Zhang, Fang Xue, Feng Lian Liu, Shu Fang Chen

**Affiliations:** 1 Department of Orthopedics, The Second Affiliated Hospital of Bengbu Medical University, Bengbu, China; 2 Nursing Department of Mingguang People's Hospital, Chuzhou, China; 3 Department of Pediatric and Preventive Dentistry, The Affiliated Stomatological Hospital of Nanjing Medical University, Nanjing, China; 4 School of Nursing, Bengbu Medical University, Bengbu, China; 5 Department of Emergency Medicine, The Second Affiliated Hospital of Bengbu Medical University, Bengbu, China

**Keywords:** emergency response competence, job burnout, mediating effect, new nurses, perceived organizational support, self-efficacy

## Abstract

**Background:**

Emergency and critically ill patients have serious illnesses that rapidly worsen and necessitate immediate medical attention, which places a great deal of demand on clinical nurses' emergency response competencies. These abilities are essential for improving nursing quality and ensuring patient safety. However, newly qualified nurses in emergency and critical care units exhibit higher levels of job burnout, which is a key risk factor influencing their emergency performance. Although other studies have shown an association between emergency response competencies and job burnout, the potential mediating roles of self-efficacy and perceived organizational support in this relationship have not been fully explored. Therefore, this study aims to examine the multiple mediating effects of perceived organizational support and self-efficacy on the relationship between job burnout and emergency response competence among new nurses in emergency and critical care departments.

**Methods:**

The study used convenience sampling to select 321 new nurses from the emergency and critical care departments of eight Grade A tertiary hospitals in Anhui Province. Data were collected through questionnaires covering general demographic information, alongside the Perceived Organizational Support Scale, Self-Efficacy Scale, Job Burnout Inventory, and Emergency Response Competence Scale for Nurses in Public Health Emergencies.

**Results:**

The total scores for job burnout, perceived organizational support, self-efficacy, and Emergency Response Competence among new nurses in emergency and critical care were 48.0 (39.0, 57.5), 41.0 (39.0, 51.0), 25.0 (20.0, 30.0), and 61.0 (54.0, 72.0), respectively. Job burnout showed negative correlations with perceived organizational support (*r* = −0.629, *P* < 0.01), self-efficacy (*r* = −0.530, *P* < 0.01), and emergency response competence (*r* = −0.560, *P* < 0.01). There was a positive correlation between perceived organizational support and self-efficacy (*r* = 0.613, *P* < 0.01) as well as emergency response competence (*r* = 0.591, *P* < 0.01). Likewise, self-efficacy also showed a positive correlation with emergency response competence. (*r* = 0.580, *P* < 0.01). Mediation analysis suggested that the indirect effect of job burnout on emergency response competence was established, with a total indirect effect value of −0.262. The independent mediating effect of perceived organizational support accounted for 30.24%, that of self-efficacy for 11.70%, and the chained mediating effect of both factors for 15.67%.

**Conclusion:**

This study indicates that newly qualified nurses in emergency and critical care settings demonstrate a moderate level of emergency response competence, which may be associated by years of work experience and environmental factors. The findings highlight that strengthening perceived organizational support and self-efficacy represents a key intervention strategy for improving emergency response competence, particularly among new nurses in emergency and critical care units who experience higher levels of job burnout. Nursing managers should focus on interventions that enhance organizational support and self-efficacy to reduce burnout and improve emergency response competence.

## 1 Introduction

Newly qualified nurses (hereafter referred to as new nurses) denote registered nurses with ≤ 3 Years' post-graduation nursing experience (1). This group commonly experiences “transition shock,” rendering them highly susceptible to job burnout during this phase (2, 3). Emergency and critical care environments, with their high workloads, significant responsibilities, and the unpredictable severity of patients' conditions, are particularly challenging. The added pressures of end-of-life care and feelings of hopelessness contribute to heightened job burnout among nursing staff (4, 5). Job burnout is defined as a three-dimensional syndrome comprising emotional exhaustion, depersonalization, and reduced personal accomplishment, arising from prolonged exposure to stressors (6). Nurse burnout stems from multiple stressors, including task overload, role conflict, interpersonal strain, and deficiencies in social support and occupational security. This phenomenon adversely affects nurses' physical and mental health, hindering their full commitment to work. It negatively impacts work behavior and attitudes, leading to reduced efficiency and diminished coping capacity, which in turn adversely affects nursing quality and patient safety (7, 8). Therefore, the hypothesis is proposed: job burnout is significantly correlated with emergency competence among new nurses in emergency and critical care settings (Hypothesis 1: job burnout → emergency response competence).

Conservation of Resource theory posits that stress arises when an individual's core resources are depleted or threatened; external support and personal characteristics serve as key mechanisms for replenishing and rebuilding these resources (9). Perceived organizational support (POS) denotes employees' overall conviction that their organization acknowledges their contributions and cares for their wellbeing–that is, the support employees perceive from their organization (10). Research confirms a negative correlation between job burnout and organizational support, with nursing managers experiencing higher perceived organizational support exhibiting lower levels of burnout (11). Building upon this, organizational support not only furnishes nurses with external resources to navigate adversity but also fortifies their proactive strategies for managing work-related stress, effectively mitigating negative emotions, and stimulating work motivation (12). Ultimately, this facilitates nurses' acquisition of the knowledge and skills required to manage diverse emergencies (13). Consequently, it is hypothesized that perceived organizational support mediates the relationship between job burnout and emergency competence among new nurses in emergency and critical care settings (Hypothesis 2: job burnout → perceived organizational support → emergency response competence).

Concurrently, self-efficacy represents an individual's confidence in achieving desired outcomes, exerting a direct influence on emergency response behaviors (14). For nurses, enhancing self-efficacy constitutes a pivotal pathway to improving their capacity for effective coping in critical situations. Relevant studies consistently confirm that heightened self-efficacy correlates positively with nurses' emergency preparedness and overall emergency response competence scores, with both indicators demonstrating significant elevation (15, 16). However, there is a notable negative correlation between job burnout and self-efficacy. When individuals experience affective depletion and reduced perceptions of achievement, their positive evaluation of personal competencies consequently declines. A study of South Korea also confirmed that there is a significant negative correlation between job burnout and self-efficacy (17). Consequently, it can be inferred that self-efficacy mediates the relationship between job burnout and emergency response competence, whereby burnout impairs emergency response by diminishing self-efficacy. (Hypothesis 3: job burnout → self-efficacy → emergency response competence).

Additionally, nursing emergency response competence refers to the ability of nursing staff to keenly observe and rapidly assess changes in a patient's condition during clinical care, and to employ proficient skills with composure and decisiveness to effectively support resuscitation and nursing interventions (18). Given the complex, unpredictable nature of critically ill patients' conditions, the urgency of treatment, and the high risks involved in nursing care, such scenarios demand heightened emergency response competence from nurses to safeguard patient safety (19). The individual-situation interaction theory posits that individual behavior arises from the combined influence of external circumstances and intrinsic personal characteristics. Consequently, emergency response competence, as an individual behavior, is related to individual motivation and competence, as well as influenced by workplace resources, such as organizational support (20, 21). This theory provides a robust framework for understanding how job burnout, perceived organizational support, and self-efficacy interact to influence emergency response competence among new nurses in critical care environments. In contrast, the Personal Emotion-Motivation Integration Model integrates several well-established theories, such as Affective Events Theory and Self-Efficacy Theory (22, 23), to explain the interaction between emotions, motivation, and behavior. While valuable for understanding this dynamic, it does not fully consider the role of external factors (e.g., organizational support). The model focuses primarily on how internal emotions and motivation influence behavior, but overlooks the impact of organizational factors in regulating emotional responses and improving performance, particularly in high-pressure environments like critical care.

**Figure 1:**
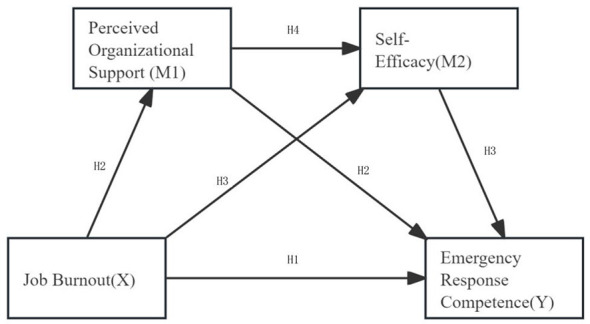
Hypothesized relationships between job burnout, perceived organizational support, self-efficacy, and emergency response competence.

Previous research has preliminarily revealed that job burnout is related to the levels of emergency response competence (8). However, the potential mediating mechanisms between these two factors—particularly the linkages centered on perceived organizational support and self-efficacy—remain unexplored among new nurses in emergency and critical care departments. (Hypothesis 4: job burnout → perceived organizational support → self-efficacy → emergency response competence). This study proposes to construct a chained mediation model to examine the influence mechanisms of perceived organizational support and self-efficacy between job burnout and emergency response competence (Figure 1). The aim is to provide a reference for nursing managers in formulating targeted intervention strategies to enhance the emergency response competence of new nurses in emergency and critical care settings.

## 2 Methods

### 2.1 Study subjects

New nurses in the emergency and critical care departments of eight Grade III Class A general hospitals within Anhui Province were selected via convenience sampling as study subjects. Data collection was conducted from April 2025 to August 2025. Inclusion criteria: 1) possession of a valid nursing registration certificate; 2) employment in emergency or ICU-related departments, including EICU, NICU, SICU, CCU, RICU, etc.; 3) ≤ 3 Years of professional experience; 4) absence of mental or physical disorders; 5) informed consent and voluntary participation. Exclusion criteria: 1) participants on sick leave or maternity leave during the study period; 2) non-clinical frontline nursing staff; 3) nursing students, rotating nurses, or trainees in emergency or ICU settings; 4) nurses temporarily assigned to emergency or ICU departments from other units. This study incorporated 20 independent variables. These comprised 14 variables for general demographic data, three dimensions of the Burnout Scale, two dimensions of the Perceived Organizational Support Scale, and one dimension of the General Self-Efficacy Scale. Based on the requirement for a sample size of at least 10 times the number of independent variables (24), and accounting for a 20% non-response rate, the minimum required sample size was 250 participants. A total of 332 questionnaires were collected in this survey. After screening for invalid responses, including those with repetitive patterns or logical inconsistencies, 321 valid questionnaires were retained, resulting in a 96.7% participation rate.

### 2.2 Research instruments

#### 2.2.1 General information questionnaire

After the researchers reviewed extensive literature and conducted discussions within the research team, this questionnaire was designed. It includes the following sections: age, gender, educational background, marital status, length of employment, reasons for pursuing nursing as a profession, satisfaction with current income, night-shift frequency, level of satisfaction with the department, clinical preceptorship model, have you had any internship or rotation experience in the Department of Emergency and Critical Care Medicine, have you undertaken specialist training in emergency and critical care medicine (excluding routine departmental training), have you ever been involved in the resuscitation of emergency and critical care patients, have you participated in emergency drills for public health emergencies.

#### 2.2.2 The job burnout inventory (Chinese version)

The Job Burnout Inventory (Chinese version) was developed by Professor Li from Henan University, China, specifically for occupational groups such as police officers, healthcare workers, and teachers (25). The scale demonstrated a Cronbach's alpha of 0.801. The scale comprises 15 items across three dimensions: emotional exhaustion (five items), depersonalization (five items), and reduced personal accomplishment (five items). Scoring employs a 7-point Likert scale ranging from “Strongly Disagree” (1) to “Strongly Agree” (7). The first two dimensions employ positive scoring, while the reduced sense of accomplishment dimension uses reverse scoring. The detection rates for each dimension of occupational burnout were calculated using the following criteria: emotional exhaustion score >25, depersonalization score >11, and reduced sense of accomplishment score >16. The Scale scores range from 15 to 105, with higher scores reflecting a greater degree of job burnout among the participants. Based on these thresholds, individuals scoring above the threshold in one dimension were classified as mildly burnt out; those scoring above the threshold in two dimensions were classified as moderately burnt out; and those scoring above the threshold in all three dimensions were classified as severely burnt out (26). In the present study, the scale demonstrated good internal consistency, with a Cronbach's alpha of 0.798.

#### 2.2.3 The Chinese emergency response competence scale

The emergency response competence of new nurses in the emergency and critical care department was assessed using the emergency response competence Scale developed by Wang et al. (27), which demonstrated a Cronbach's alpha of 0.950. The scale is composed of three distinct dimensions: emergency knowledge (5 items), emergency response competence (4 items), and comprehensive capability (9 items). Each item employed a five-point Likert scale, scored from 1 (very poor) to 5 (very good), yielding a total score ranging from 18 to 90 points. Higher scores indicate superior emergency response competence, while lower scores denote inferior capability. The mean score across all items serves as the evaluation benchmark: a mean score < 3 indicates low emergency response competence; a mean score of 3–4 indicates moderate capability; and a mean score >4 indicates high capability. In this study, the scale demonstrated high internal consistency, with a Cronbach's alpha of 0.913.

#### 2.2.4 Perceived organizational support scale for nurses

The Nurses' Perceived Organizational Support Scale, developed by Zuo (28), was employed to investigate the perceived organizational support among new nurses in the Emergency and Critical Care units. The internal consistency reliability for this scale was confirmed, as indicated by a Cronbach's alpha of 0.90. It comprises two distinct dimensions: affective support, measured by 10 items, and instrumental support, evaluated through three items. Responses were recorded on a 5-point Likert format (1 = strongly disagree, 5 = strongly agree), yielding aggregate scores between 13 and 65. A total score ≤ 38 indicated low organizational support perception, while >38 signified high organizational support perception. Higher scores reflected stronger organizational support perception among participants. The scale showed high internal consistency (Cronbach's α = 0.893) in the current study.

#### 2.2.5 General self-efficacy scale

The General Self-Efficacy Scale (GSES) was employed to measure the self-efficacy levels of new nurses in the emergency and critical care settings. This scale was developed by Professor Ralf Schwarzer and colleagues from Germany (29). The Chinese adaptation of the General Self-Efficacy Scale was initially developed and validated by Zhang et al., with reported internal consistency reliability (Cronbach's α) reaching 0.87 (30). The scale is unidimensional, comprising 10 items. It employs a four-point Likert format, with item responses scored from 1 (“completely incorrect”) to 4 (“completely correct”), yielding aggregate scores between 10 and 40. According to the scores, three levels can be identified: low (ranging from 10 to 20), moderate (21 to 30), and high (31 to 40), with increasing scores reflecting stronger levels of self-efficacy. The scale showed high internal consistency (Cronbach's α = 0.874) in the current study.

### 2.3 Data collection

This study strictly adheres to the ethical principles outlined in the Declaration of Helsinki and has been approved by the Ethics Committee of Bengbu Medical University [Ethics Approval No. (2025) 579]. Before commencing data collection, authorization was obtained from the nursing departments of all participating hospitals. A cross-sectional survey was conducted by two uniformly trained postgraduate students utilizing the Questionnaire Star platform. First, inclusion and exclusion criteria were clearly defined. The questionnaire's introductory page detailed the study's objectives, significance, and completion requirements using standardized guidance text, emphasizing voluntary participation, anonymity, and confidentiality principles. Following informed consent from all participants, the nurse manager distributed the questionnaire link via departmental WeChat groups. To safeguard data quality, the system was configured to permit only one submission per IP address and WeChat account, with all questions designated as mandatory.

### 2.4 Statistical analysis

Analysis was conducted using SPSS 27.0 statistical software. Count data were described using frequency and percentage; non-normally distributed continuous data were described using median and interquartile range. Differences in emergency response capability scores among new nurses with varying characteristics in critical care settings were analyzed using non-parametric rank-sum tests. Correlations between job burnout, perceived organizational support, self-efficacy, and emergency response competence were assessed using Spearman's correlation analysis. After ensuring the residuals of the dependent variable satisfied the four requirements, “linearity”, “independence”, “homogeneity of variance”, and “normal distribution”, multiple linear regression analysis was employed to examine the factors influencing emergency response competence. The Process macro model 6 was used to test chained mediating effects, with a significance level of α = 0.05.

## 3 Results

### 3.1 Common method bias test

Using Harman's single-factor test, exploratory factor analysis was conducted on all items of the Job Burnout Scale, Emergency Response Competence Scale, Perceived Organizational Support Scale, and Self-Efficacy Scale. The results revealed 15 factors with eigenvalues >1. The first common factor accounted for 30.79% of the total variance, which is below the 40% critical threshold, confirming no significant common method bias.

### 3.2 General information

The demographic characteristics of 321 new nurses are as follows: Gender: male 115 (35.8%), female 206 (64.2%). Age: ≤ 25 years old, 120 (37.4%); 26–30 years old, 188 (58.6%); >30 years old, 13 (4.0%). Education: associate degree or below, 94 (29.3%); bachelor's degree, 190 (59.2%); master's or above, 37 (11.5%). Marital status: unmarried, 198 (61.7%); married, 123 (38.3%). Reasons for pursuing nursing as a profession: passion, 85 (26.5%); livelihood, 166 (51.7%); parental expectation, 58 (18.1%); other, 12 (3.7%). Satisfaction with current income: dissatisfied, 69 (21.5%); neutral, 158 (49.2%); satisfied, 94 (29.3%). Night-shift frequency (shifts/month): 0, *n* = 11 (3.4 %); 1–4, *n* = 48 (15.0%); 5–9, *n* = 205 (63.9%); ≥10, *n* = 57 (17.8%). Clinical preceptorship model: no preceptor, *n* = 90 (28.0 %); fixed one-to-one mentor, *n* = 61 (19.0%); fixed group of senior mentors, *n* = 50 (15.6 %); shift-based dynamic mentor assignment, *n* = 120 (37.4 %). Prior internship or rotation in emergency and critical care: none, *n* = 101 (31.5 %); yes, *n* = 220(68.5%). No statistically significant differences (*P* > 0.05) were observed in emergency response competence scores among new critical care nurses across these characteristics. Statistically significant differences in emergency response competence scores were observed for the following factors (*P* < 0.05): length of employment, level of satisfaction with the department, participation in critical care-specific training, involvement in critical patient resuscitation, and engagement in public health emergency drills, as shown in Table 1.

**Table 1:** Comparison of emergency response competence scores among new nurses in emergency and critical care settings with different characteristics (*n* = 321).

Variable	*n* (%)	Emergency response competence score Median (Q1, Q3)	*Z/H*	*P*
Length of employment (years)			*H* = 24.258	< 0.001
≤ 1	55 (17.1)	54 (43, 59)		
>1 and ≤ 2	97 (30.2)	62 (54, 78)		
>2 and ≤ 3	169 (52.6)	63 (55, 72)		
Level of satisfaction with the Department			*H* = 10.481	0.005
Dissatisfied	107 (33.3)	62 (56, 75)		
Neutral	102 (31.8)	59.5 (54, 68)		
Satisfied	112 (34.9)	62.5 (46.25, 72)		
Have you undertaken specialist training in emergency and critical care medicine?			*Z* = −2.808	0.005
No	76 (23.7)	58 (53, 66.75)		
Yes	245 (76.3)	62 (54, 73)		
Have you ever been involved in the resuscitation of emergency and critical care patients?			*Z* = −7.371	< 0.001
No	31 (9.7)	40 (38, 50)		
Yes	290 (90.3)	62 (54, 72)		
Have you participated in emergency drills for public health emergencies?			*Z* = −7.089	< 0.001
No	102 (31.8)	54 (44.75, 63)		
Yes	219 (68.2)	66 (56, 76)		

### 3.3 Scores for job burnout, perceived organizational support, self-efficacy, and emergency response competence among new nurses in emergency and critical care departments

The total scores for Job burnout, perceived organizational support, self-efficacy, and Emergency Response Competence among new nurses in emergency and critical care departments were 48.0 (39.0, 57.5) points, 41.0 (39.0, 51.0) points, 25.0 (20.0, 30.0) points, and 61.0 (54.0, 72.0) points, respectively, as shown in Table 2.

**Table 2:** Scores of job burnout, perceived organizational support, self-efficacy, and emergency response competence among new nurses in emergency and critical care departments [Median (Q1, Q3), points; *n* = 321].

Variable	Total score	Entry score
Job burnout	48.0 (39.0, 57.5)	3.2 (2.6, 3.8)
Emotional exhaustion	20.0 (17.0, 26.5)	4.0 (3.4, 5.3)
Depersonalization	9.0 (6.0, 11.0)	1.8 (1.2, 2.2)
Reduced personal accomplishment	17.0 (12.0, 20.0)	3.4 (2.4, 4.0)
Perceived organizational support	41.0 (39.0, 51.0)	3.2 (3.0, 3.9)
Emotional support	30.0 (27.0, 39.0)	3.0 (2.7, 3.9)
Instrumental support	12.0 (9.0, 12.0)	4.0 (3.0, 4.0)
Self-efficacy	25.0 (20.0, 30.0)	2.5 (2.0, 3.0)
Emergency response competence	61.0 (54.0, 72.0)	3.4 (3.0, 4.0)
First-aid skills	15.0 (12.0, 17.0)	3.8 (3.0, 4.3)
Emergency knowledge	16.0 (15.0, 20.0)	3.2 (3.0, 4.0)
Comprehensive competence	30.0 (27.0, 36.0)	3.3 (3.0, 4.0)

### 3.4 Correlation among job burnout, perceived organizational support, self-efficacy, and emergency response competence

Spearman correlation analysis revealed that burnout was negatively correlated with perceived organizational support, self-efficacy, and emergency response competence (*r* = −0.629 to −0.530, all *P* < 0.01) among the new nurses in emergency and critical care departments. Perceived organizational support shows significant positive associations with both self-efficacy and emergency response competence (*r* = 0.591 to 0.613, all *P* < 0.01). Additionally, self-efficacy was positively correlated with emergency response competence (*r* = 0.580, *P* < 0.01), as shown in Table 3.

**Table 3:** Correlation analysis of job burnout, perceived organizational support, self-efficacy, and emergency response competence among new nurses in emergency and critical care departments (*r* values).

Variable	Job burnout	Perceived organizational support	Self-efficacy	Emergency response competence
Job burnout	1			
Perceived organizational support	−0.629^**^	1		
Self-efficacy	−0.530^**^	0.613^**^	1	
Emergency response competence	−0.560^**^	0.591^**^	0.580^**^	1

^**^P < 0.01.

### 3.5 Multiple linear regression analysis of emergency response competence among new nurses in emergency and critical care departments

Emergency response competence was set as the dependent variable. Independent variables included those showing statistical significance in the univariate analysis and those demonstrating significant correlations in the Spearman correlation analysis. After confirming that the residuals of the dependent variable met the four assumptions of linearity, independence, homoscedasticity, and normal distribution, multiple regression analysis was performed to identify influencing factors (*P* < 0.05). Before the regression analysis, variables were assigned as follows: continuous variables were entered as original values, binary and ordinal variables were assigned numerical values, and unordered categorical variables were dummy-coded (see Table 4). The results indicated that length of employment, experience in rescuing critically ill patients, participation in emergency drills for public health incidents, job burnout, perceived organizational support, and self-efficacy were significantly related to emergency response competence among new nurses in emergency and critical care departments (*P* < 0.05). The model statistics were as follows: *R*^2^ = 0.552, adjusted *R*^2^ = 0.540; *F* = 21.49, *P* < 0.001, indicating that the model was statistically significant. Tolerance values ranged from 0.413to 0.971 (all >0.1), and variance inflation factor (VIF) values ranged from 1.030 to 2.419 (all < 5), suggesting no severe multicollinearity among the variables, as shown in Table 5.

**Table 4:** Assignment of independent variables.

Variable	Variable name	Assignment notes
Length of employment	X1	1 = ≤ 1,2 = >1 and ≤ 2,3 = >2 and ≤ 3
Level of satisfaction with the department	X2	1 = Dissatisfied,2 = Neutral,3 = Satisfied
Have you undertaken specialist training in emergency and critical care medicine?	X3	0 = NO,1 = YES
Have you ever been involved in the resuscitation of emergency and critical care patients?	X4	0 = NO,1 = YES
Have you participated in emergency drills for public health emergencies?	X5	0 = NO,1 = YES
Job burnout	X6	Entered as original values
Perceived organizational support	X7	Entered as original values
Self-efficacy	X8	Entered as original values

**Table 5:** Multiple linear regression analysis of emergency response competence among new nurses in emergency and critical care departments.

Variable	β	SE	Beta	*t*	*p*	95% CI
(Constant)	30.069	5.852		5.138	< 0.001	18.555 to 41.583
Length of employment	2.008	0.796	0.107	2.524	0.012	0.443 to 3.574
Level of satisfaction with the department	0.282	0.663	0.016	0.425	0.671	−1.023 to 1.588
Have you undertaken specialist training in critical care medicine?	−1.222	1.358	−0.036	−0.9	0.369	−3.895 to 1.450
Have you ever been involved in the resuscitation of emergency and critical care patients?	9.051	2.101	0.188	4.308	< 0.001	4.917 to 13.186
Have you participated in emergency drills for public health emergencies?	4.103	1.307	0.134	3.139	0.002	1.531 to 6.674
Job burnout	−0.211	0.056	−0.195	−3.762	< 0.001	−0.321 to −0.100
Perceived organizational support	0.293	0.074	0.234	3.976	< 0.001	0.148 to 0.438
Self-efficacy	0.589	0.127	0.245	4.636	< 0.001	0.339 to 0.839

r = 0.743; R^2^ = 0.552; adjusted R^2^ = 0.540; F = 21.49; P < 0.001.

### 3.6 Analysis of the mediating effects of perceived organizational support and self-efficacy on the relationship between job burnout and emergency response competence

**Figure 2:**
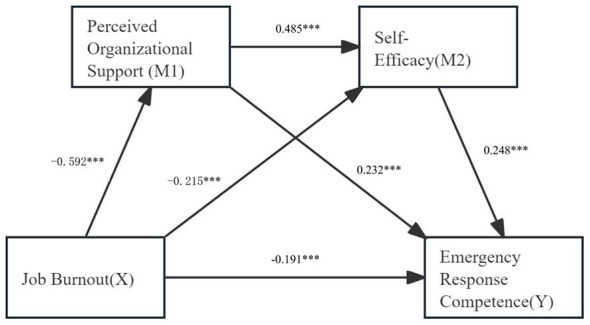
Chain mediation model. ***P < 0.001.

A chain mediation analysis was conducted using Model 6 in the PROCESS macro, with emergency response competence as the dependent variable, burnout as the independent variable, and perceived organizational support and self-efficacy as mediators. Variables that showed statistical significance in the multiple linear regression analysis (length of employment, experience in rescuing critically ill patients, and participation in emergency drills for public health incidents) were included as covariates. The mediation effect was tested by repeatedly sampling 5,000 times using the Bootstrap sampling method. The results suggested that perceived organizational support mediated the relationship between job burnout and emergency response competence, with a mediation effect value of −0.137. Similarly, self-efficacy acted as a mediator between job burnout and emergency response competence, with a mediation effect value of −0.053. Furthermore, perceived organizational support and self-efficacy exhibited a chain-mediated effect between job burnout and Emergency Response Competence, with a chain mediation effect value of −0.071. The 95% confidence intervals for all three paths do not include zero, suggesting statistical significance and indicating the chained mediating effect. The effect sizes for these three pathways accounted for 30.24%, 11.70%, and 15.67% of the total effect, respectively. The total indirect effect accounted for 57.84% of the total effect. Details are presented in Table 6, and the mediation model is illustrated in Figure 2.

**Table 6:** Analysis of the mediating effects of perceived organizational support and self-efficacy between job burnout and emergency response competence.

Path Effect	β	Boot SE	Bootstrap 95%CI	Percent (%)
Total effect	−0.453	0.043	−0.539 to −0.368	100.00
Direct effect	−0.191	0.052	−0.293 to −0.090	42.16
Indirect effect	−0.262	0.042	−0.342 to −0.175	57.84
X → M1 → Y	−0.137	0.044	−0.216 to −0.044	30.24
X → M2 → Y	−0.053	0.020	−0.095 to −0.018	11.70
X → M1 → M2 → Y	−0.071	0.022	−0.119 to −0.033	15.67

X = job burnout; M1 = perceived organizational support,; M2 = self-efficacy; Y = emergency response competence.

## 4 Discussion

### 4.1 Relationship and current status of job burnout and emergency response competence among new nurses in emergency and critical care departments

#### 4.1.1 Relationship between job burnout and emergency response competence

This study found that burnout is significantly related to the emergency response competence of new nurses in emergency and critical care departments, with a mediation effect value of −0.191, accounting for 42.16% of the total effect. This confirms Hypothesis 1. During the transition from university to clinical practice, new nurses encounter abrupt shifts in role, responsibilities, environment, and interpersonal relationships. Multiple factors—including lack of clinical experience, the gap between idealized expectations and reality, work-life imbalance, and disrupted social support—can readily precipitate burnout (31–33). Within the “high workload-high stress-high risk” environment of critical care, job burnout has been demonstrated to constitute a significant threatening stressor for both the work performance and mental health of new nurses (4). Without effective support and intervention, these compounding pressures further erode their work motivation and confidence, which is significantly related to their emergency response competence (34, 35).

#### 4.1.2 Analysis of the current state of job burnout

This study included 321 newly qualified nurses in emergency and critical care, with a job burnout prevalence rate of 67.0%. This finding is significantly higher than the 27.1% reported by Bruyneel et al. (36) and the 43.2% reported by Gong et al. (37). This indicates a more pronounced level of job burnout among this group, which is potentially related to the specificity of the study population. This study focuses exclusively on new nurses in emergency and critical care departments. For this group, transition shock is a universal phenomenon that makes them highly susceptible to job burnout. Furthermore, this cohort operates within the high-load, high-risk clinical environment of critical care departments, where patients present with complex and rapidly changing conditions. This demands exceptional professional competence and emergency response competence from nursing staff, which may be associated with diminished personal accomplishment and occupational burnout under intense work stress. Nurse burnout is significantly related to patient safety and the mental health of nursing staff themselves (38, 39). In light of this, nursing managers must prioritize addressing job burnout among new critical care nurses, implementing early identification and intervention to safeguard patient safety and uphold the physical and mental wellbeing of nursing staff.

#### 4.1.3 Analysis of the current state of emergency response competence

According to the emergency response competence grading criteria (27), this study suggests that the emergency response competence of new nurses in emergency and critical care settings is at a moderate level, which is lower than findings from previous research (40, 41). This may be related to their relatively short professional tenure, during which they are still accumulating knowledge and skills. As this cohort primarily undertakes foundational nursing duties, they often face challenges in responding effectively to the sophisticated equipment and unpredictable, critical patient conditions characteristic of emergency and critical care settings. In contrast, mid-career and senior nurses, defined as those with 11 or more years of work experience, have encountered more emergencies and participated in the management of more critically ill patients, thereby accumulating richer clinical experience. They demonstrate greater composure, clearer role division, and more orderly management when responding to emergency incidents (18, 42). The survey revealed that new nurses participated in critical patient resuscitations and public health emergency drills significantly less frequently than mid-career and senior nurses. While their emergency capabilities are passable, there remains considerable scope for improvement. It is recommended that nursing management departments enhance systematic, simulation-based emergency training for new nurses in emergency and critical care settings. Combining case-based teaching with practical drills will elevate their emergency response and management capabilities during clinical emergency incidents.

### 4.2 Mediating role of perceived organizational support

This study reveals that perceived organizational support plays a crucial mediating role between job burnout and emergency response competence, with an effect size of−0.137. This mediating effect accounts for 30.24% of the total effect. The mediation effect of 30.24% in this study is notably higher than the 14% reported by Chen, who studied clinical nurses with over 6 years of experience in tertiary hospitals (21). In contrast, our study focused on new nurses in emergency and critical care units with ≤ 3 years of experience, facing different work environments and higher stress. This likely explains the larger mediation effect observed here. Given the high-stress, high-risk conditions new nurses work in, a 30.24% mediation effect for perceived organizational support can be considered strong. Chen's study, involving more experienced nurses in less stressful settings, found a more modest effect. These findings underscore the important role of organizational support in mitigating burnout and improving emergency response competence among new nurses in critical care. Specifically, job burnout among new nurses in emergency and critical care units not only has a direct negative relationship with their emergency response competence but also appears to be indirectly related to it through perceived organizational support. Consequently, Hypothesis 2 is validated. A finding consistent with the “resource depletion spiral” mechanism outlined by the Conservation of Resources (COR) Theory. When individuals face sustained resource depletion, they enter a defensive mode, exhibiting defensive or withdrawal behavioral responses (9, 43). This manifests as reduced sensitivity to external situational cues and diminished positive evaluation of available resources, thereby inhibiting their perception and utilization of organizational support (44). More precisely, burnout not only diminishes individuals' subjective perception of organizational contextual resources (such as emotional support, information provision, and instrumental support) but further obstructs their pathways to accessing and integrating external supportive resources, thereby exacerbating the decline in emergency abilities (44, 45). In other words, elevated levels of job burnout among new nurses are related to a decreased perception of organizational support, which is associated with a reduction in their coping capacity. This conclusion is consistent with previous research (46), which indicates that healthcare professionals with higher perceived levels of organizational support demonstrate superior emergency response competence. Accordingly, it is recommended that hospital administrators construct an organizationally supportive environment through multiple dimensions, including emotional care, work support, and value recognition, to systematically enhance new nurses' perception of organizational support. By activating the “resource acquisition spiral” mechanism, individuals can strengthen their loyalty to the organization and professional commitment during the process of obtaining external resource compensation, thereby enabling them to reciprocate with higher levels of engagement and performance in clinical emergency practice.

### 4.3 Mediating role of self-efficacy

This study demonstrates that self-efficacy partially mediates the relationship between job burnout and emergency response competence, with an effect size of −0.053. The mediating effect accounts for 11.70% of the total effect. These findings indicate that job burnout among new nurses in emergency and critical care units is significantly related to their emergency response competence and may also be indirectly related to it through the mediating effect of self-efficacy, thereby supporting Hypothesis 3. When compared to Lee's study, which reported a 20.65% mediating effect of self-efficacy, the mediating effect observed in this study appears to be lower. Lee's research was conducted during the COVID-19 pandemic with infection control nurses, who, due to their extensive experience and professional competence, may have had higher confidence when facing high levels of pressure, responsibility, and workload. This could explain the higher mediating effect observed in Li's study. In contrast, this study focuses on new nurses in emergency intensive care units. Although they face considerable work pressure, their lack of experience and confidence in managing the complex tasks of emergency critical care may hinder the development of effective coping mechanisms, as seen in more experienced nurses. This could account for the smaller mediating effect observed in this study. Research further confirms that job burnout is negatively associated with self-efficacy (17). However, enhancing self-efficacy significantly has been found to improve emergency confidence and behavior (47). Specifically, high-load, high-risk clinical settings induce emotional exhaustion and diminished personal accomplishment among new nurses at the psychological load level. This subsequently weakens their psychological resources, reducing confidence and perceived control in emergency scenarios (manifesting as helplessness, passive behavior, and avoidance of decision-making), which may affect the effective execution of their emergency responses. These findings align with Bandura's self-efficacy theory (23), where beliefs about one's capabilities significantly influence motivation, emotional regulation, and ultimate performance. Based on this, new nurses can be guided to develop a positive evaluation of both their circumstances and self-perception when encountering stress and negative emotions, regarding daily work pressures as opportunities to accumulate professional experience and foster personal growth. Consequently, nursing managers are advised to address new nurses' occupational burnout while simultaneously elevating their self-efficacy through systematic training, simulation exercises, and psychological support. This dual approach fortifies their emergency confidence and capabilities in critical care scenarios, thereby securing both patient safety and nursing quality.

### 4.4 The chain mediation effect of perceived organizational support and self-efficacy

Within the macro framework of the Individual-Situation Interaction Theory, this study confirms the chain-mediating role of perceived organizational support and self-efficacy in the relationship between job burnout and emergency response competence,. The standardized effect size was −0.071, with the mediating effect accounting for 15.67% of the total effect. Hypothesis 4 was thus validated. The demanding nature of nursing duties in critical care departments requires a combination of professional knowledge, clinical skills, and effective teamwork. New nurses, often lacking clinical experience and facing challenges in collaboration, are particularly vulnerable to psychological exhaustion in high-stress, high-risk environments, leading to job burnout. This aligns with the Conservation of Resources theory, which posits that individuals with depleted resources (such as energy, confidence) are more prone to burnout when faced with persistent stressors (9). In addition, The findings of this study are consistent with the Job Demands-Resources theory (48), where job demands (such as workload and stress) activate personal resources (like self-efficacy) and external resources (such as organizational support) to enhance work motivation and performance. Findings support the notion that strengthening nurses' organizational support and self-efficacy can mitigate the negative effects of burnout on emergency response capabilities. This is consistent with prior research, which suggests that improving nurses' organizational support and self-efficacy can boost their work engagement and resilience in challenging environments (49). From the perspective of the Individual-Situation Interaction Theory, this study highlights how the interaction between situational resources (organizational support) and personal resources (self-efficacy) creates a synergistic effect. This combined influence is crucial in enhancing new nurses' emergency response capabilities in critical care settings. This finding is theoretically significant as it shows that the interaction between organizational and personal resources not only buffers burnout but also improves work ability under stress, extending the application of the existing model to high-risk emergency and critical care environments. In light of these findings, hospital management should consider implementing a comprehensive support system, including emotional, instrumental, and informational support, to foster nurses' organizational belonging and professional confidence. By doing so, new nurses will be better equipped to handle complex tasks in high-risk departments, improving their psychological wellbeing and performance in critical care situations.

## 5 Limitations

Firstly, this study employed convenience sampling to recruit 321 newly qualified nurses in emergency and critical care from eight Grade A tertiary general hospitals in Anhui Province. While this approach was practical, it may limit the representativeness of the sample, as the hospitals selected may not fully represent the diversity of healthcare settings across other regions in China, particularly in remote areas where healthcare access and resources may differ. This limits the external validity of the findings. Future research should aim to use multi-center stratified sampling across different provinces and both urban and rural areas to improve the generalizability and representativeness of the findings. Secondly, the use of self-reporting questionnaires for data collection may introduce subjective reporting bias, as participants may be influenced by social desirability, privacy concerns, or personal perceptions when responding to the questions. This could result in biased data, affecting the accuracy of the results. Future studies could combine self-report measures with objective data collection methods, such as performance assessments or peer evaluations, to enhance the reliability of the findings. Furthermore, the cross-sectional design of this study constrains causal inference among the variables. The relationships observed in this study are correlational, and future research should adopt a longitudinal design with multi-center stratified sampling to validate causal sequencing, assess the robustness of effects, and explore the potential influencing factors.

## 6 Conclusion

This study adopted the PROCESS macro (Model 6) to test the multiple mediating pathways of job burnout on emergency response competence, confirming the existence of three transmission mechanisms: (1) the independent mediating effect of perceived organizational support; (2) the independent mediating effect of self-efficacy; and (3) the chain mediating effect from perceived organizational support to self-efficacy. It further indicated that job burnout among new nurses in emergency and critical care settings is associated with a reduction in their emergency response competence. These findings reveal the combined influence of external contexts and individual intrinsic traits on the relationship between job burnout and emergency response competence, thereby enriching the individual-situation interaction theory framework within nursing contexts. Based on these findings, nursing managers should adopt targeted interventions, including: (1) Tailored Training Programs: focused on building both technical and emotional resilience skills for new nurses in critical care settings. (2) Peer Support Systems: establishing mentorship programs to reduce burnout and enhance organizational support. (3) Confidence-Building Initiatives: implementing simulation-based training and regular feedback to improve confidence in handling complex tasks. (4) Workload Management: distributing tasks equitably and rotating shifts to manage work pressure and prevent burnout. These interventions aim to reduce job burnout and enhance emergency response competence, which could contribute to improved patient safety and the wellbeing of new nurses in emergency and critical care settings.

## Data Availability

The raw data supporting the conclusions of this article will be made available by the authors, without undue reservation.
